# Diagnosis and treatment of hereditary angioedema with normal C1 inhibitor

**DOI:** 10.1186/1710-1492-6-15

**Published:** 2010-07-28

**Authors:** Konrad Bork

**Affiliations:** 1 Department of Dermatology, Johannes Gutenberg University, Mainz, Germany

## Abstract

Until recently it was assumed that hereditary angioedema is a disease that results exclusively from a genetic deficiency of the C1 inhibitor. In 2000, families with hereditary angioedema, normal C1 inhibitor activity and protein in plasma were described. Since then numerous patients and families with that condition have been reported. Most of the patients by far were women. In many of the affected women, oral contraceptives, hormone replacement therapy containing estrogens, and pregnancies triggered the clinical symptoms. Recently, in some families mutations in the coagulation factor XII (Hageman factor) gene were detected in the affected persons.

## Introduction

Angioedema is clinically characterized by self-limiting episodes of marked edema involving the skin, gastrointestinal (GI) tract and other organs. Various forms of acquired and hereditary angioedema (HAE) share this clinical presentation. "Classic" HAE is associated with a quantitative (type I) or qualitative (type II) deficiency of C1 esterase inhibitor (C1-INH) caused by mutations of the C1-INH gene. Until recently it was assumed that HAE is a disease that results exclusively from a genetic deficiency of the C1-INH. In 2000, 10 families with this disease were described [1]. In these families a total of 36 women, but not a single man, were affected. All patients had normal C1-INH concentration and activity with respect to C1 esterase inhibition, ruling out both types of HAE (HAE type I and HAE type II). This hitherto unknown disease was proposed to be termed as "hereditary angioedema with normal C1 inhibitor occurring mainly in women" or "hereditary angioedema type III." Subsequently, two additional families were described, with seven affected women in one family and four in the other [2,3]. Later on, clinical data on an additional 29 women with HAE type III were presented [4]. Because all 76 patients from the studies cited above were women, it was assumed that the clinical phenotype might be limited to the female sex. However, in 2006 a family with dominantly inherited angioedema and normal C1-INH was described in which not only five female but also three male family members were clinically affected [5]. Later on, a number of further patients with HAE type III were reported [6-10].

In 2001 the author of this article initiated a microsatellite scan of the total genom (performed by Dr. C. Hennies, Max-Delbrück Center, Berlin) in four HAE type III families which revealed major linkage signals for chromosomes 6 and 16 but not for chromosome 5 (unpublished data). By following a functional hypothesis that the genetic defect might be located in the coagulation factor XII (FXII) gene the factor XII gene on chromosome 5 was then selectively investigated [11]. In May 2006, the causative genetic mutations in 6 index patients of 20 families and in 22 patients of the corresponding 6 families were identified: two different missense mutations have been verified which were responsible for the disease according to the co-segregation pattern (see below) [11]. The location of these mutations is the same locus, 5q33-qter of the Hageman factor or coagulation FXII gene (Online Mendelian Inheritance in Man # 610619). One mutation leads to a threonine-to-lysine substitution (Thr309Lys) and the other to a threonine-to-arginine substitution (Thr309Arg). The mutations were located on the exon 9. It was also found that the index patients of 14 further families with HAE and normal C1-INH did not show these mutations (see below) [11]. So the 2 mutations in the factor XII gene could be found only in some families with HAE type III and not in others.

Hence, the following types of HAE can be differentiated today: (a) hereditary angioedema due to a genetic C1-INH deficiency (HAE-C1-INH) including type I and type II; and (b) hereditary angioedema with normal C1-INH (HAE type III) including hereditary angioedema due to the two known mutations in the coagulation factor XII gene (HAE-FXII) and hereditary angioedema with an unknown genetic cause (normal C1-INH activity in plasma, no causative mutation in the gene coding for C1-INH and none of the known FXII gene mutations Thr309Lys or Thr309Arg) (HAE-unknown).

## Clinical presentation

### Clinical symptoms

The clinical symptoms of HAE with normal C1-INH include: recurrent skin swellings, abdominal pain attacks, tongue swellings, and laryngeal edema. Until now, only a relatively small number of patients and families have been described. In 2000, it was reported that 36 patients exhibited relapsing skin swellings and/or attacks of abdominal pain and/or recurrent laryngeal edema [1]. Urticaria did not occur at any time in any of these patients. The skin swellings lasted 2-5 days; they affected mainly the extremities and the face, and the trunk less frequently. The abdominal attacks likewise lasted 2-5 days and were manifested as severe cramp-like pains. In a more recent study, a total of 138 patients with HAE with normal C1-INH who belonged to 43 unrelated families were examined [12]. A majority of patients had skin swellings (92.8%), tongue swellings (53.6%), and abdominal pain attacks (50%). Laryngeal edema (25.4%) and uvular edema (21.7%) also were frequent, whereas edema episodes of other organs were rare (3.6%). Facial swellings and tongue involvement occurred considerably more frequently compared with HAE-C1-INH. The number of patients with recurrent edema of only one organ was higher than in HAE-C1-INH. Erythema marginatum was not observed. Hence, HAE with normal C1-INH levels shows a characteristic pattern of clinical symptoms. There are many differences in the clinical symptoms and course of disease between this type of HAE and the classic type of HAE, HAE-C1-INH (Appendix 1).

The clinical manifestation of HAE type III is highly variable, and penetrance of the disease might be low; thus, obligate female carriers, even in their seventh decade, without any clinical symptoms were observed [1,4]. Therefore, a considerable number of asymptomatic carriers may exist in the population.

### Death by asphyxiation due to upper airway obstruction

In a patient series described in 2007[12] one female had asphyxiated at the age of 16 during her first laryngeal edema attack. A second female asphyxiated at the age of 36 after 10 episodes of upper airway obstruction, a third at the age of 38 during her eighth airway attack, and a fourth at the age of 48 after a tongue swelling.

### Onset of clinical symptoms

In a series of 138 patients, the mean age at onset of the disease was 26.8 years (SD+/- 14.9 years, range 1 to 68 years) [12]. Onset of clinical symptoms occurred in the first decade of life in 11 (8%) patients, in the second decade in 60 (43.5%) patients, in the third decade in 22 (15.9%) patients, and later in 45 (32.6%) patients. Hence, the number of patients with disease onset in adulthood was significantly higher in HAE with normal C1-INH compared with HAE-C1-INH.

## Potentially provoking factors

### 1. Role of estrogens

In many women clinical symptoms either begin or are exacerbated following the intake of oral contraceptives or hormone replacement therapy, or during pregnancy [1-4]. This observation has led to the assumption that the clinical manifestation of this new type of HAE is estrogen-dependent. Binkley and Davis observed patients with typical symptoms of recurrent angioedema that were restricted to conditions of high estrogen levels and thereby created the conception of an "estrogen-dependent" or "estrogen-associated" HAE [2,13]. However, in an analysis of 228 angioedema patients receiving oral contraceptives or hormone replacement therapy, it was demonstrated that in only 24 (62%) of 39 women with HAE type III were the clinical symptoms induced or exacerbated after starting oral contraceptives or hormone replacement therapy; correspondingly, 15 (38%) of 39 women tolerated exogenous estrogens without any influence on their disease [4]. Almost identical numbers were observed with respect to women diagnosed with HAE-C1-INH. These results show that estrogens play a role in both conditions and that the negative influence of estrogens is not a specific sign for HAE type III [14].

### 2. Angiotensin-converting enzyme inhibitors

It is well known that angiotensin-converting enzyme inhibitors (ACE-I) are associated with the occurrence of angioedema in about 0.7% of individuals who receive this medication [15,16]. It has been reported that ACE-I can induce an exacerbation of symptoms in patients with HAE-C1-INH [17]. A 60-year-old man from a family with HAE with normal C1-INH was reported who has had arterial hypertension since age 30 and had four tongue swellings following treatments with captopril and enalapril [5]. The last episode occurred when the patient received only hydrochlorothiazide and metoprolol. The patient has had no other symptoms of HAE. This observation demonstrates that ACE-I might have a trigger function with regard to HAE type III. HAE type III shares this feature with HAE-C1-INH. This state of affairs points to an important role of bradykinin in the pathogenesis of HAE type III (see below).

### 3. Angiotensin II type 1 receptor antagonists

Two unrelated patients with preexisting HAE type III were described who experienced severe exacerbation of symptoms associated with using angiotensin II type 1 receptor antagonists (angiotensin II type 1 receptor blockers, ARB) [18]. A possible pathogenetic relationship between the underlying disease and the drug-associated angioedema was suggested.

## Gender

The disease has been observed predominantly by far in women [1-4,11,12]. In two families, however, the existence of clinically unaffected male carriers has been deduced [2,3]. In 2006 a family with dominantly inherited angioedema and normal C1 inhibitor was described in which not only five female but also three male family members were clinically affected [5]. Later on further male patients with HAE type III were reported, among them also patients with HAE-FXII [8,12]. The familial angioedema observed by Gupta et al.[19] in three brothers appears to be a HAE with normal C1-INH in men; however, a possibly recessive inheritance pattern and a favorable response to treatment with antihistamines may indicate that the three brothers' condition is different from that of the family we observed [5]. In a study of 25 patients with idiopathic nonhistaminergic angioedema, Cicardi et al.[20] mentioned that four of these patients had affected relatives. In at least three of these families, all affected individuals were male.

## Inheritance

Within the 43 families described in 2007[12], between two and 10 members per family were affected. The examination of the pedigrees of the 43 families revealed that 2 successive generations were affected in 30 families, 3 successive generations were affected in 9 families, and 4 successive generations were affected in 4 families. These results support the assumption of a dominant inheritance pattern.

## Genetic results

C1-INH activity and C4 in plasma were normal in most patients and slightly decreased in a small proportion of patients [12,14]. Therefore, right from the beginning it seemed to be improbable that the cause of the disease would be a mutation in the C1-INH gene. Binkley and Davis [2] found no abnormalities in either the 5' regulatory region or the coding sequences of the C1-INH gene in affected individuals. In four of our affected patients we also looked for mutations in the C1-INH gene and did not find any. Binkley and Davis also sequenced the 5' regulatory region of factor XII gene because it contains a known estrogen response element. However, they found no abnormalities in that region.

In 2006, a genetic examination revealed new insight into HAE type III (see above) [11]. It was hypothesized that an abnormal coagulation factor XII molecule may lead to inappropriate activation of the kinin-forming cascade of which factor XII is a major constituent. Therefore, a search for mutations in the factor XII (Hageman factor) (*F12*) gene was performed [11]. In 20 unrelated patients with HAE type III the 14 exons and splice junctions of the *F12 *gene were screened by PCR amplification and bidirectional sequencing. Two different non-conservative missense mutations were identified in exon 9. Both mutations are located in exactly the same position, namely in the second position of the codon (ACG) encoding amino acid residue 309 of the mature protein, a threonine residue. One mutation, encountered in five unrelated patients, results in an AAG triplet encoding a lysine residue (Thr309Lys). The other mutation, observed in one patient, predicts a threonine-to-arginine substitution (Thr309Arg). Thus, with respect to both mutations, the wild-type threonine residue is substituted by a basic amino acid residue. In accordance with the dominant inheritance pattern of the disease, patients are heterozygous for the respective mutations. Neither of the two mutations was detected in 145 healthy control individuals in this control panel. In six of the 20 families, 20 individuals, all female, were clinically diagnosed with HAE with normal C1-INH. All these 20 women were found to be heterozygous carriers of either the Thr309Lys or the Thr309Arg mutation. Two additional women carried the Thr309Lys mutation but have not experienced any angioedema symptoms up to now. Finally, there were eight male heterozygous carriers of a missense mutation of Thr309, all symptom-free [11].

Until now, the Thr309Lys mutation has been reported in 11 families surveyed in our Angioedema Outpatient Service [11,14] and in 8 families studied by other authors, one family by each of them [6-8,21-25].

## Potential role of the mutations in the FXII gene in HAE-FXII

The predicted structural and functional impact of the mutations in the factor XII gene, their absence in healthy controls, and their co-segregation with the phenotype all provide strong support for the idea that these mutations cause disease. The remarkable observations that (1) two different mutations seen in patients but not controls both affect the identical DNA position, and (2) both lead to substitution of the wild-type threonine residue by a positively charged residue, lend further support to the assumption that these mutations play a disease-causing role.

It is not clear how the mutations in the FXII gene cause HAE-FXII, i.e. the tendency to develop recurrent and self-limiting edema attacks in various organs. There are several arguments for the assumption that the kallikrein-kinin system (KKS) also known as the "contact system" or "contact activation system" might be involved in the pathogenesis: (a) the causative mutations are in the FXII gene, and FXII is part of KKS; (b) KKS activation with the release of bradykinin at the end of the cascade is known to cause the acute attacks of HAE due to C1-INH deficiency; and (c) corticosteroids and antihistamines are therapeutically ineffective for the treatment of swelling in HAE-FXII, therefore, histamine does not seem to play a major role in HAE-FXII.

Coagulation factor XII is a serine protease circulating in human plasma as a single-chain inactive zymogen at a concentration of approximately 30 μg/ml [26-29]. Upon contact with negatively charged surfaces, factor XII is activated by autoactivation and by plasma kallikrein, which itself is generated from prekallikrein by activated factor XII, high-molecular weight kininogen serving as a co-factor for reciprocal activation of factor XII and prekallikrein. Factor XII is a typical mosaic protein: following a leader peptide of 19 residues, the mature plasma protein consists of 596 amino acids and is organized in an N-terminal fibronectin type-II domain, followed by an epidermal growth factor-like domain, a fibronectin type-I domain, another epidermal growth factor-like domain, a kringle domain, a proline-rich region, and the C-terminal catalytic serine protease domain [27]. The described amino acid substitutions are located in the poorly characterized proline-rich region of factor XII [11]. This region appears to play some role in the binding of factor XII to negatively charged surfaces [28,29]. Thus, one may speculate that those mutations may influence mechanisms of contact activation and may eventually inappropriately facilitate factor XII activation.

A report of patients with HAE-FXII demonstrated a more than 4-fold increase in FXIIa amidolytic activity on S-2302 compared with healthy controls [6]. The increased enzymatic activity was blocked completely by 2 mM PCK, and the report stated that PCK specifically inhibits FXII activation in human plasma. Based on these findings, it was suggested that the FXII Thr309Lys mutation (referred to as Thr328Lys by adding the leader protein) is a gain-of-function mutation that markedly increases FXII amidolytic activity but that does not alter FXII plasma levels [6]. In a more recent study, elements of the kallikrein-kinin system and the downstream-linked coagulation, complement, and fibrinolytic systems in the plasma of six patients with HAE caused by the Thr309Lys mutation and healthy probands were examined [30]. The mean FXII clotting activity was 90% in patients with the FXII mutation and the concentration of FXIIa was 4.1 ng/ml; this did not differ from healthy probands. Mean prekallikrein amidolytic activity and high-molecular weight kininogen clotting activity were 130% and 144%, respectively, both higher than in healthy probands. The mean kallikrein-like activity of the HAE patients was 11.4 U/l and did not differ from the healthy probands. There was no difference in FXII surface activation by silicon dioxide or in kallikrein-like activity with and without activation by dextran sulfate. Contrary to the results of the study mentioned before [6] no indication that the Thr309Lys mutation causes a "gain-of-function" of FXIIa was observed in this investigation. Hence, the functional role of the observed FXII gene mutations in HAE type III still remains unclear.

The mediator responsible for edema formation in HAE type III is not known. However, consider the following facts: (a) there are many similarities concerning clinical symptoms of hereditary angioedema types I and III; (b) the percentages of women whose disease is negatively affected by estrogen-containing medications is similar in both conditions; (c) angiotensin-converting enzyme inhibitors and angiotensin II type 1 receptor antagonists may lead to an increase in frequency and severity of attacks in HAE type III (according to the observations mentioned above) similar to HAE due to C1 inhibitor deficiency (HAE type I and II); and (d) the response to antihistamines and corticosteroids is lacking, at least in the patients reported up to now. These facts permit the speculation that edema formation in HAE type III may also be related to the kinin pathway. It is possible that bradykinin is the most important mediator in HAE type III, similar to HAE type I and II.

## Diagnosis

Up to now the clinical diagnosis of "hereditary angioedema with normal C1 inhibitor" has required that patients have the above-mentioned clinical symptoms, one or more family members also affected with these symptoms, the exclusion of familial and hereditary chronic urticaria with urticaria-associated angioedema, and normal C1-INH activity and protein in plasma. The diagnosis "hereditary angioedema with coagulation factor XII gene mutation" (HAE-FXII) requires the corresponding demonstration of the mutation. Until now there is no further laboratory test which could confirm the diagnosis "HAE type III".

The question whether there are sporadic, non-familial cases cannot be answered satisfyingly today. Sporadic cases of HAE-FXII with no mutations in the FXII gene in the close relatives have not been reported until now. To identify sporadic cases of HAE-unknown is not possible at present since there are no laboratory tests available to confirm the diagnosis of this subtype of HAE III (see also under "idiopathic angioedema", see below).

## Differential diagnosis

The most important differential diagnosis of HAE type III are other types of recurrent angioedema. Angioedema is a clinical sign that belongs to various clinical entities. Some of them are due to a hereditary or acquired C1-INH deficiency such as HAE types I and II and acquired angioedema due to C1-INH deficiency. Other types are not associated with a C1-INH deficiency. Besides HAE with normal C1-INH (HAE type III) they include angioedema due to ACE-I and ARB, angioedema associated with an urticaria, allergic or non-allergic angioedema caused by insect stings, food or certain drugs, and idiopathic angioedema.

### (a) Hereditary angioedema due to C1 inhibitor deficiency

In HAE, edema of the skin, abdominal pain attacks, and life-threatening laryngeal edema are the most frequently encountered symptoms, their relation being 70:54:1 [31,32]. Skin swellings occur most frequently in the extremities and less frequently on the face or on other body sites [33]. Abdominal attacks of HAE are mostly characterized by pain, vomiting and diarrhea. They are caused by transient edema of the bowel wall, leading to partial or complete intestinal obstruction, ascites, and hemoconcentration. C1-INH activity and C4 protein are low in plasma. The features of HAE type III that serve to differentiate it from hereditary angioedema due to C1-INH deficiency are listed is Appendix 1.

### (b) angioedema due to angiotensin converting enzyme inhibitors and angiotensin-II receptor blockers

ACE-I are commonly used for the treatment of hypertension and congestive heart failure. Recurrent angioedema as a complication of therapy with ACE-I is well described in the literature [15,16]. Most often it occurs as a well-demarcated swelling of the tongue, lips, or other parts of the face. Edema of the mucous membranes of the mouth or throat are less frequently. Isolated dysphagia or edema of the gastrointestinal tract are rare. Angioedema of the upper respiratory tract can result in acute respiratory distress, airway obstruction and, rarely, death. Angioedema due to ACE-I often occurs within one week after starting the medication. Numerous patients, however, have been reported in whom the first angioedema occurred after some weeks or months or even years after starting treatment with ACE-I. Patients with a history of recurrent idiopathic angioedema may be at increased risk for developing ACE-I induced angioedema. The ARB which exert their antihypertensive effect through specific blockade of the angiotensin II via blockade of the angiotensin subtype 1 receptor appears to have a much lower incidence of recurrent angioedema. The history of hypertension or congestive heart failure and the onset of recurrent angioedema following the treatment with ACE-I and the non-familial occurrence separates ACE-I-induced angioedema clearly from HAE type III.

### (c) Urticaria-associated angioedema

More than 50% of patients with a chronic urticaria have one or more episodes of angioedema in their history of urticaria. So in those patients angioedema seems to be a part of chronic urticaria [34]. In most patients by far, chronic urticaria and urticaria-associated angioedema respond to antihistamines. Patients with HAE type III have only angioedema and no urticaria as far as it is known today. Furthermore HAE type III usually does not respond to antihistamines. So the differentiation of urticaria-associated angioedema from HAE type III can clearly be made by patients' history and by clinical features.

### (d) Allergic or non-allergic angioedema caused by insect stings, food, certain drugs, or infections

Insect stings, the intake of certain food or certain drugs may lead to allergic (anaphylactic) or nonallergic (anaphylactoid) reactions [35,36]. Mainly they include urticaria, angioedema, and circulatory reactions due to a drop of blood pressure reaching from faintness to severe shock. Each of these symptoms may occur alone or they may occur together in various combinations. In this context one or more episodes of isolated angioedema may occur, mostly as a facial swelling. These are reactive swellings, i.e. swellings with a recognizable trigger. They do not occur without a trigger. Therefore they can be clearly separated from HAE type III.

### (e) Idiopathic angioedema

This type of angioedema is poorly understood. Already the definition of idiopathic angioedema varies considerably. Some authors include urticaria-associated angioedema [34], others restrict the diagnosis of idiopathic angioedema to patients with recurrent angioedema without an urticaria. It is a fact that there is a number of patients with recurrent angioedema which cannot be classified to one of the types of recurrent angioedema mentioned above, despite an extensive diagnostic workup. Probably, recurrent idiopathic angioedema without urticaria is not a single disease. Three types of idiopathic angioedema were proposed: one type in which patients responded to antihistamines (idiopathic histaminergic angioedema), another one without a response to antihistamines but response to tranexamic acid (idiopathic nonhistaminergic angioedema), and a third type not responding to both antihistamines and tranexamic acid [20,35].

HAE type III is defined as a hereditary disease; in all families reported until now more than one individual per family were affected. Whether there are sporadic, nonfamilial cases is presently not known. Patients with HAE-FXII and no other family member with mutations in the FXII gene would have a new mutation. Such patients have not been reported until now. Whether some of the patients with idiopathic angioedema are solitary cases of HAE-unknown cannot be proven since at present there are no labaratory tests available for diagnosing this subtype of HAE type III.

## Management

### Treatment of Acute Attacks

Until now, acute attacks of HAE type III were treated with a C1-INH concentrate, icatibant, corticosteroids, antihistamines, and adrenalin (Table 1). In one study, 7 patients with HAE-XII received a C1-INH concentrate (Berinert^®^, CSL Behring, Inc., Marburg, Germany) for 63 angioedema attacks [14]. One patient who received this agent once for an abdominal attack reported that it was not effective. In the other 6 patients, the agent was very or moderately effective. Recently, 3 patients with HAE type III were reported who were treated with icatibant, a bradykinin B2 receptor antagonist used in Europe for acute attacks of HAE-C1-INH [37]. Time to resolution of symptoms was 1 to 2 hours in the 3 treated attacks. In one attack the symptoms recurred after 6 hours and necessitated a second injection of icatibant. In 23 of our patients with HAE type III [1], previous angioedema attacks had been treated with corticosteroids (at a dosage of 100-250 mg one or more times daily) and antihistamines; however, this treatment was ineffective in all 23 cases. Likewise, in other studies [10,14,38] patients with HAE type III did not respond to corticosteroids and antihistamines.

**Table 1:** Treatments in hereditary angioedema with normal C1-INH (HAE type III) as reported up to now

	Treatment effective (no. of patients; [reference])	Treatment not effective (no. of patients; [reference])
**Acute attacks**		

C1-INH concentrate	6 [14]	4 [1]
		1 [14]

Icatibant	3 [37]	0

Corticosteroids	0	23 [1]
		1 [7]
		1 [22]

Antihistamines	0	23 [1]
		1 [7]
		1 [21]
		1 [22]

Adrenalin	0	1 [21]

**Prophylaxis**		

Androgens	2 [1]	0
	1 [3]	
	1 [38]	

Tranexamic acid	1 [10]	2 [1]
	1 [14]	1 [37]

Progesterone	1 [5]	0
	1 [10]	
	8 [14]	

### Prophylactic Treatment

Progesterone, danazol, and tranexamic acid have been used prophylactically to prevent angioedema attacks (Table 1). In one study, 8 patients with HAE-FXII received a progesterone-containing and estrogen-free oral contraceptive [14]. Seven of these patients took desogestrel, which is a progestagen, for 1 to 6 years, for a total of 27 years. One of these 7 patients was switched to an implant with etonogestrel for 3 years. The remaining woman received injections of medroxyprogesteron for 3 years. The 8 women were symptom-free during progesterone treatment. One woman with HAE-FXII received danazol (200 mg), an attenuated androgen, daily for 12 years [14]. While on treatment, she was symptom free. During these 12 years, she discontinued danazol twice. Each cessation of treatment was followed by a series of severe abdominal attacks, tongue swellings, and skin swellings, and each time the patient resumed treatment. To date, the patient has had no side effects from danazol treatment. A second patient who received danazol (100 mg) daily for 6 years for severe HAE symptoms was also free of symptoms during treatment. The dose was subsequently tapered and discontinued; during the 2 years between discontinuation and the present, no HAE symptoms were observed [14]. Other studies [3,38,39] have also shown an improvement in symptoms in patients with HAE type III during treatment with danazol. One woman with HAE-FXII who started tranexamic acid therapy (4 g/d) has had no attacks with this treatment regimen [14].

## Conclusions

Hereditary angioedema with normal C1 inhibitor (HAE type III) is clinically characterized by recurrent angioedema affecting the skin, gastrointestinal tract, and larynx. Skin swellings are the most frequent symptoms of HAE type III. Most often they occur on the face, less frequently at the extremities, and only in rare cases at the genitals. Tongue swellings and abdominal pain attacks are less frequent symptoms. Laryngeal edema is rare. Death by asphyxiation as a result of attacks of upper airway obstruction has been observed. Women are more often affected than men. In some women the clinical symptoms of HAE type III occur exclusively in periods of oral contraceptives, hormonal replacement therapy or pregnancies, indicating that estrogens may have a considerable influence on the phenotypical disease expression. Only limited data on the molecular basis of HAE type III are currently available. In some families with HAE and normal C1-INH, mutations in the FXII gene have been found in the affected patients. The cosegregation of these mutations with the disease phenotype demonstrates the causative role of the mutations. Several treatment options are available for HAE type III, including C1-INH agents, progesterone, danazol, and tranexamic acid.

## Abbreviations

ACE-I: angiotensin-converting enzyme inhibitors; ARB: angiotensin II type 1 receptor blockers; C1-INH: C1 esterase inhibitor; DNA: deoxyribonucleic acid; FXII: coagulation factor XII; HAE: hereditary angioedema; HAE-C1-INH: hereditary angioedema due to C1 inhibitor deficiency; HAE-FXII: hereditary angioedema due to mutations in the factor XII gene; KKS: kallikrein-kinin system.

## Competing interests

KB discloses that he is a speaker for CSL Behring, Shire and Viropharma.

## Appendix 1. Features of hereditary angioedema with normal C1-INH that serve to differentiate it from hereditary angioedema due to C1-INH deficiency

• Patients have normal C1-INH protein and activity.

• Mainly women are clinically affected.

• The number of children already affected before the age of 10 years is low. Clinical symptoms start in adulthood in more patients than in hereditary angioedema due to C1-INH deficiency.

• There are more disease-free intervals during the course of the disease.

• Symptoms are less frequent compared with hereditary angioedema due to C1-INH deficiency.

• Facial swellings, mainly lip swellings, are relatively more frequent.

• The tongue is considerably more often affected: Recurrent tongue swelling is observed in many patients and is a cardinal symptom of the condition.

• Many patients have only skin swellings.

• Many patients have only recurrent skin swellings and tongue swellings.

• Abdominal attacks are less frequent.

• Suffocation may be preceded and caused by a tongue swelling.

• There is no erythema marginatum (gyrated erythematous rash) as is highly characteristic of HAE due to C1-INH deficiency.

• Hemorrhages into skin swellings were observed in hereditary angioedema with normal C1-INH.
